# Early diagnosis of Alzheimer’s disease from elevated olfactory mucosal miR-206 level

**DOI:** 10.1038/srep20364

**Published:** 2016-02-04

**Authors:** Jangsup Moon, Soon-Tae Lee, Il Gyu Kong, Jung-Ick Byun, Jun-Sang Sunwoo, Jung-Won Shin, Ji-Young Shim, Ji-Hyun Park, Daejong Jeon, Keun-Hwa Jung, Ki-Young Jung, Dong-Young Kim, Sang Kun Lee, Manho Kim, Kon Chu

**Affiliations:** 1 Department of Neurology, Laboratory for Neurotherapeutics, Comprehensive Epilepsy Center, Biomedical Research Institute, Seoul National University Hospital, Seoul, South Korea; 2 Program in Neuroscience, Seoul National University College of Medicine, Seoul, South Korea; 3 Advanced Neural Technologies, Seoul, South Korea; 4 Department of Otorhinolaryngology-Head and Neck Surgery, Hallym University Sacred Heart Hospital, Hallym University College of Medicine, Anyang, South Korea; 5 Department of Neurology, CHA University College of Medicine, Seoungnam, South Korea; 6 Protein Metabolism Medical Research Center, Seoul National University College of Medicine, Seoul, South Korea; 7 Department of Otorhinolaryngology-Head and Neck Surgery, Seoul National University Hospital, Seoul National University College of Medicine, Seoul, South Korea

## Abstract

MicroRNA-206, which suppresses the expression of brain-derived neurotrophic factor, is known to be elevated in the brains of Alzheimer’s disease (AD) patients. We performed intranasal biopsy of the olfactory epithelia of early dementia patients (n = 24) and cognitively healthy controls (n = 9). Patients with significant depression (n = 8) were analyzed separately, as their cognitive impairments were thought to be caused by their depression. Real-time PCR was performed on the biopsied tissues. The relative microRNA-206 level exhibited a 7.8-fold increase (P = 0.004) in the mild cognitive impairment group (CDR 0.5; n = 13) and a 41.5-fold increase (P < 0.001) in the CDR 1 group (n = 11). However, this level was not increased in the depression group, even in those with cognitive decline. Using the optimal cutoff value, the sensitivity/specificity for diagnosing CDR 0.5 and CDR 1 dementia were 87.5%/94.1% and 90.9%/93.3%, respectively. In ROC analysis, the AUCs were 0.942 and 0.976 in the CDR 0.5 and CDR 1 groups, respectively. The olfactory mucosal microRNA-206 level and cognitive assessment scores were significantly correlated in the non-depressed subjects with cognitive impairment. In conclusion, the olfactory mucosal microRNA-206 level can be easily measured, and it can be utilized as an excellent biomarker for the diagnosis of early AD, including mild cognitive impairment.

More than 35 million people worldwide are currently suffering from Alzheimer’s disease (AD), and the number of patients will increase to >100 million by 2050^1^. Unfortunately, no single medication showing disease-modifying effects has been approved by the US Food and Drug Administration to date^2^,^3^. Therefore, the early diagnosis of patients in the incipient stage of AD is essential for patient care and also to plan clinical trials for potential AD drugs. However, most AD diagnoses are based on clinical findings^4^. Multiple biomarkers have been suggested, but there are limitations associated with each biomarker, and most of them have been shown to be ineffective in differentiating individuals with mild cognitive impairment (MCI) from cognitively healthy people^5^. Consequently, there is still an unmet need for a better biomarker capable of the very early detection of AD.

Brain-derived neurotrophic factor (BDNF) is the most widely expressed neurotrophin in the central nervous system, and it has a neurotrophic effect on neurons by binding to its specific receptor, tyrosine receptor kinase B^6^. Moreover, BDNF is a key molecule involved in the maintenance of synaptic plasticity and synaptogenesis in the hippocampus, which is the core locus of memory acquisition and consolidation^7^,^8^. AD is considered to be caused by synaptic failure^9^, and decreased BDNF levels have been reported in the brain and blood of AD patients and AD animal models^6^. A low serum BDNF level has been associated with a smaller hippocampal volume and poorer memory^10^. Additionally, the serum BDNF level has been negatively correlated with the severity of dementia^11^, and the Val66Met polymorphism of the BDNF gene has been proposed to be a marker for the prediction of disease progression in MCI patients^12^. Recently, it has been reported that BDNF may also be reduced in healthy people who are destined to develop dementia or AD^13^.

MicroRNAs (miRNAs) are regulators of numerous biological processes, and alterations in miRNA levels occur in various human diseases^14^. miRNA-206 (miR-206) is conventionally known as one of the myomiRs, which are abundant in muscle tissue and play important roles in muscle development and muscle remodeling^15^. However, recent studies have shown that miR-206 also has regulatory roles in other mammalian non-muscle tissues, including tumor suppressor functions in various types of cancer^16^. In a previous study, we have shown that miR-206 is elevated in the brains of AD patients and animal models and that it contributes to cognitive decline by suppressing BDNF expression in the brain (Fig. 1)^17^. However, there is no method available to evaluate the altered expression of a specific miRNA in the brain of a living subject. Recently, the olfactory epithelium (OE) has been receiving attention for its potential use in the research of neurodegenerative disease, as it reflects pathological changes in the brain^18^,^19^,^20^.

In this study, we investigated whether miR-206 is elevated in the OE of early AD patients and whether the olfactory mucosal miR-206 level is an appropriate biomarker for the diagnosis of early AD.

## Results

### Basic characteristics of the subjects

A total of 41 subjects were enrolled in our study. The subjects were categorized based on their clinical dementia rating (CDR) score and Beck Depression Inventory-II (BDI-II) score. Nine patients who were cognitively healthy and did not exhibit depression were assigned to the CDR 0 (control) group. In addition, thirteen patients were assigned to the CDR 0.5 group, and eleven were assigned to the CDR 1 group. Eight patients with moderate to severe depression were placed in the depression group. The ages of the patients varied across the groups (P = 0.039; Table 1).

Increased miR-206 levels in the olfactory epithelia of patients with early dementia. 

**Figure 2:**
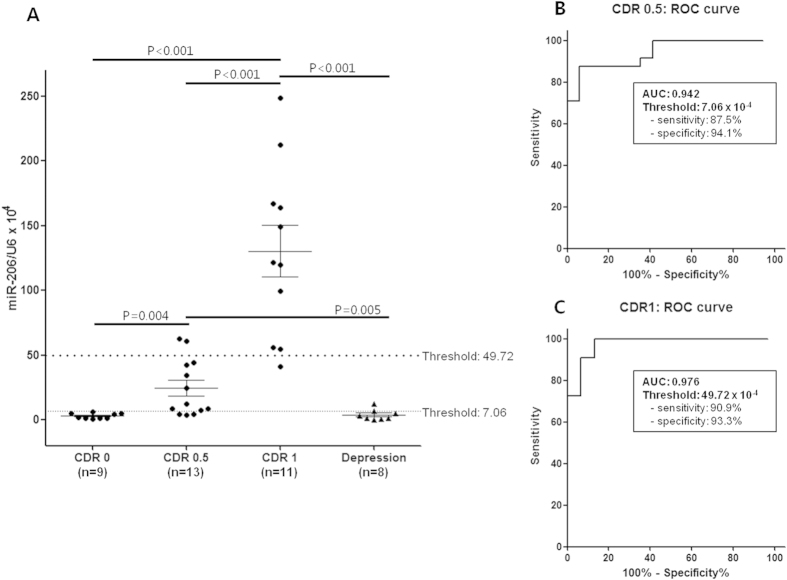
miRNA-206 real-time PCR results for olfactory epithelial samples. Panel **A** demonstrates the relative miRNA-206 level (normalized to the U6 level) in each patient. The two cutoff values were determined by receiver operating curve (ROC) analysis, which revealed the highest sum of sensitivity and specificity for the diagnosis of CDR 0.5 or CDR 1 dementia from the entire population. The diagnosis of CDR 0.5 in ROC analysis signifies the detection of patients with a CDR ≥0.5. Panels **B** and **C** depict the results of ROC analyses for the diagnosis of CDR 0.5 and CDR 1 dementia, respectively. The areas under the curve (AUCs) are 0.942 and 0.976 for CDR 0.5 and CDR 1 dementia, respectively.

The miR-206 level was increased in the early dementia group compared with that in the control group (CDR 0.5: 7.8-fold increase, *P* = 0.004; CDR 1: 41.5-fold increase, *P* < 0.001; Fig. 2A), and it showed a remarkable tendency to increase with the progression of dementia. The patients in the depression group did not exhibit a significant alteration in the miR-206 level (1.2-fold increase, *P* = 0.671; Fig. 2A), although 6 of the 8 patients complained of cognitive dysfunction (Supplementary Table S1).

Receiver operating characteristic (ROC) analysis was performed, and the effectiveness of the olfactory mucosal miR-206 level for the diagnosis of CDR 0.5 or CDR 1 dementia was assessed in the entire patient population. A diagnosis of CDR 0.5 signifies the detection of patients with a CDR ≥ 0.5. The optimal cutoff values for detecting CDR 0.5 or CDR 1 patients were selected. An area under the curve (AUC) value of 0.942 indicated a diagnosis of CDR 0.5 dementia, and when the relative expression of miR-206 over U6 exceeded 7.06 × 10^−4^, the sensitivity was 87.5% (95% CI, 69.0–95.7) and the specificity was 94.1% (95% CI, 73.0–99.0; Fig. 2B). Only one patient in the depression group exhibited a higher miR-206 level than this cutoff value; however, this patient also complained of cognitive decline, with a CDR of 0.5 (Supplementary Table S1). None of the cognitively healthy patients had a miR-206 level of higher than this value (Fig. 2A). An AUC value of 0.976 indicated a diagnosis of CDR 1 dementia, and if the relative expression of miR-206 over U6 exceeded 49.72 × 10^−4^, the sensitivity was 90.9% (95% CI, 62.3–98.4) and the specificity was 93.3% (95% CI, 78.7–98.2; Fig. 2C). Two of the 13 (15.4%) patients with CDR 0.5 had a higher miR-206 level than this cutoff value, and none of the individuals with CDR 0 or depression exhibited a miR-206 level that exceeded this value (Fig. 2A). Additionally, ROC analysis was performed specifically on the non-depressed cognitive impairment patients. The olfactory mucosal miR-206 level was also useful for identifying the progression of dementia, with an AUC value of 0.944 for the diagnosis of CDR 1 dementia (Supplementary Fig. S1).

Considering early-onset AD is defined as onset before the age of 65, we assessed the ability of the olfactory mucosal miR-206 level to differentiate the presence of early dementia among patients younger than 65 years old versus those older than 65 years. Regardless of the age population, the miR-206 levels were significantly different among the groups. The diagnostic value of the olfactory mucosal miR-206 level was found to be greater among the patients who were younger than 65 years old. The patients with CDR 0.5 and CDR 1 demonstrated 11.9-fold and 57.1-fold increases in the miR-206 levels, respectively, compared with the control group (Supplementary Table S2). The AUC values for diagnosing CDR 0.5 and CDR 1 dementia were 0.952 and 1.000, respectively (Supplementary Fig. S2A,.B,C). The olfactory mucosal miR-206 level also demonstrated a fairly good ability to detect early dementia in the patients older than 65 years of age, although with less power than that observed for the patients younger than 65 years of age (Supplementary Table S2; Supplementary Fig. S2D,E,F).

### Correlation of olfactory mucosal miR-206 level with clinical scores

**Figure 3:**
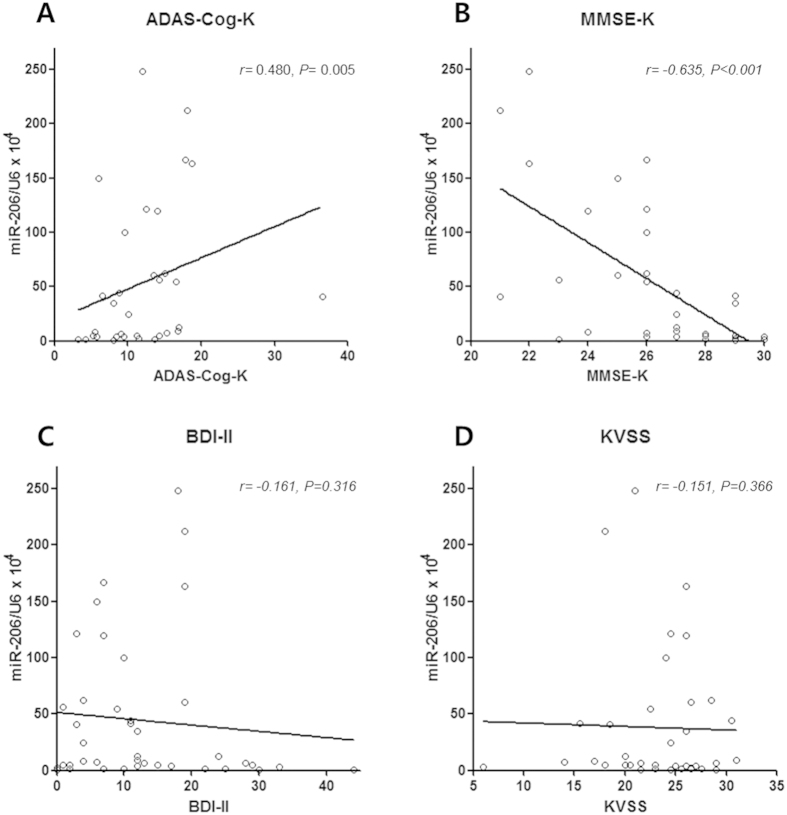
Correlation analyses between olfactory mucosal miR-206 level and clinical measures. The correlations between the olfactory mucosal miR-206 level and cognitive assessment measures were analyzed in the non-depressed subjects. As shown in panels A and B, the olfactory mucosal miR-206 level is well correlated with the ADAS-Cog-K (P = 0.005) and MMSE-K scores (P < 0.001). As shown in panels C and D, the olfactory mucosal miR-206 level in all patients is not correlated with the BDI-II (P = 0.316) or KVSS score (P = 0.366). Abbreviations: ADAS-Cog-K: Korean version of the Alzheimer’s Disease Assessment Scale-cognitive subscale, MMSE-K: Korean version of the Mini-Mental State Examination, BDI-II: Beck Depression Inventory-II, KVSS: Korean version of the Sniffin’ Stick Test.

As depression is known to affect cognitive function, we assessed the correlation between the relative miR-206 level and the cognitive function scores, exclusively in the patients without depression (n = 33). A significant correlation was detected between the olfactory mucosal miR-206 level and these scores. The relative miR-206 level was positively correlated with the Korean version of Alzheimer’s Disease Assessment Scale-cognitive subscale score (ADAS-Cog-K score; r = 0.480, P = 0.005; Fig. 3A) and negatively correlated with the Korean version of the Mini-Mental State Examination score (MMSE-K; r = −0.635, P < 0.001; Fig. 3B). The correlations among the BDI-II score, the Korean version of the Sniffin’ Stick Test (KVSS) score and the relative miR-206 level were assessed for all patients. The relative miR-206 level was not found to be correlated with the BDI-II score (Fig. 3C) or KVSS score (Fig. 3D).

### Safety of olfactory epithelial biopsy

Most episodes of nasal bleeding were controlled within 30 minutes after biopsy. Only one participant was taking antiplatelet agents at the time of biopsy. All of the participants returned home without any acute complications, including the one who was taking low-dose aspirin. We called each participant the next day to check for any complaints and informed them to contact us whenever they experienced any discomfort. Four of the 41 individuals (9.8%) experienced minor complications. Two of them complained of sustained nasal bleeding and had to return to the clinic. However, after additional hemostasis, they did not experience any further epistaxis. One patient complained of a mild headache, and another experienced watery rhinorrhea after biopsy, which naturally subsided within a few days. The remaining patients did not encounter any discomfort after the procedure.

## Discussion

This is the first study to demonstrate the overexpression of miR-206 in the OE of patients with early dementia. The olfactory mucosal miR-206 level exhibited a sharp increasing tendency as dementia progressed, and it was significantly elevated, even in the patients with MCI (CDR 0.5 group; >7-fold increase), and was immensely increased in the patients in the CDR 1 group (>41-fold increase). However, the expression of olfactory mucosal miR-206 was not altered in the patients with depression, even in those with combined cognitive impairment. The relative miR-206 level in the OE could be a promising new biomarker for the early detection of AD, even at the MCI stage, and for the identification of subjects who might benefit from a miRNA-based treatment for AD.

Early diagnosis of AD is essential for patient care and for the success of AD drug trials. However, the diagnosis of AD still relies upon clinical decision making. Despite rapid advances in the identification of CSF markers and multiple types of neuroimaging, most current strategies are insufficient for differentiating MCI patients from cognitively healthy people^5^,^21^. The biomarkers for AD can be classified into two broad categories: (1) markers of amyloid-β (Aβ) accumulation; and (2) markers of neuronal injury or neurodegeneration^21^,^22^,^23^. The CSF Aβ42 assay and amyloid positron emission tomography (PET) imaging belong to the first category^24^; however, amyloid positivity does not reliably indicate clinical diagnosis. Although amyloid positivity on amyloid PET is reported in 90% of patients who are clinically diagnosed with AD dementia, 60% of patients with MCI and 30–40% of cognitively intact older people also exhibit amyloid deposits in the brain^25^. CSF phosphorylated tau (p-tau) 181 and total tau levels and 18F-fluorodeoxyglucose (FDG)-PET are markers of neuronal injury or neurodegeneration^26^,^27^. These markers help to predict progression to dementia in subjects with MCI^28^, but alterations are already present in many clinically normal elderly patients, making it impossible to distinguish normal aging from MCI or AD^5^,^25^.

The brain miR-206 level is elevated in patients with AD, and this miRNA may participate in the core pathogenesis of cognitive decline^17^. It has been reported that miR-206 post-transcriptionally suppresses BDNF expression^29^,^30^. BDNF is among the key regulators of synaptic plasticity and memory, and AD brains exhibit a decreased level of BDNF, which might be responsible for the cognitive impairment^7^. Several experiments have been performed with the aim of increasing the brain BDNF level in AD animal models^31^,^32^, but further study is necessary. Our group has reported for the first time that miR-206 is overexpressed in AD brains, both in animal models and in humans. We demonstrated that the brain BDNF level can be increased by antagonizing miR-206 activity, which eventually leads to the improvement of cognitive function^17^. The role of miR-206 in the regulation of brain BDNF was subsequently reproduced in later studies^33^,^34^. Therefore, the detection of patients with elevated brain miR-206 is important not only for making an early diagnosis of AD but also for identifying proper candidates for future AD drug trials based on the modulation of miR-206.

The OE, which is pseudostratified columnar epithelium located on the roof of the superior nasal cavity, is known to reflect pathological changes and gene expression profiles of the brain in association with many neurological diseases, including Parkinson’s disease^20^, schizophrenia^35^, and AD^19^. The OE of schizophrenia patients has been reported to demonstrate pathological evidence of abnormal neurodevelopment^36^,^37^. AD pathology, amyloid-β and abnormal tau protein have been reported to be present in the OE in the majority of patients with advanced AD^38^. The OE can be safely and easily biopsied in live human subjects via an intranasal approach^39^; thus, it has gained attention for its potential utility as a surrogate tissue in the research of brain disorders^40^.

In addition to its easy accessibility, the OE can reflect dynamic changes in the brain and can be used as a dynamic marker. Sattler *et al*. have demonstrated pharmacodynamic changes in the OE after drug administration^41^. Altered miRNA expression is known to be involved in the pathogenesis of many neurodegenerative diseases^42^. When it becomes possible to detect temporal changes in the levels of pathogenic miRNAs in the brain, this approach will be an excellent means to identify patients in the incipient stage of disease. Recently, Mor *et al*. have demonstrated that miR-382 expression is increased in the OE of schizophrenia patients, and this miRNA might be utilized as a novel biomarker of schizophrenia in living patients^35^.

The olfactory mucosal miR-206 level may be an excellent biomarker for the diagnosis of early AD for several reasons. First, it is significantly increased from the MCI stage; thus, it is effective for diagnosing patients with incipient AD. It is remarkable that the patients in the CDR 0 group had uniformly low olfactory mucosal miR-206 levels, while those in the CDR 0.5 group exhibited >7-fold increased levels. At the optimal cutoff value, the relative miR-206 level demonstrated fairly good sensitivity (87.5%) and specificity (94.1%) for the diagnosis of CDR 0.5 dementia. It is noteworthy that the cutoff value demonstrated greater specificity than sensitivity. This excellent specificity will help in the selection of patients in the very early stage of AD with minimal false positives. False positive results have been the major limitations of previous AD biomarkers being considered for clinical use^5^. The inclusion of many patients in the very early stage of AD with the minimization of false positive cases will improve the success rates of clinical trials. Notably, among the individuals who were under age 65, who are the subjects of early-onset AD by definition, the CDR 0.5 group demonstrated a larger increase (11.9-fold increase) in the miR-206 level and greater specificity (100%) at the optimal cutoff value. Accordingly, the olfactory mucosal miR-206 level will be a valuable screening tool for the selection of incipient AD patients who might be enrolled in AD drug trials.

Second, the olfactory mucosal miR-206 level showed excellent sensitivity and specificity for diagnosing CDR 1 dementia. At the optimal cutoff value, the sensitivity was 90.9% and the specificity was 93.3%. In ROC analysis, the AUC value was 0.976, which is much higher than those of previous biomarkers of AD. When the subjects were restricted to non-depressive cognitive impairment patients, the AUC value was 0.944, which suggests that the level of this miRNA can effectively indicate the progression of MCI. Many previous ROC analyses using single or combined biomarkers have yielded AUC values that were lower than our results (CSF Aβ42: 0.810–0.928, CSF t-tau: 0.80–0.911, CSF p-tau: 0.753–0.880, CSF t-tau/Aβ42: 0.85–0.917, p-tau/Aβ42: 0.84, FDG-PET: 0.88, multiple biomarkers: –0.942)^23^,^43^,^44^,^45^. Achievement of an excellent AUC value using a single biomarker is an important finding.

Third, the olfactory mucosal miR-206 level can be measured to differentiate AD from cognitive impairment related to depression. In the patients without significant depression, the level of this miRNA was strongly correlated with the ADAS-Cog-K and MMSE-K scores. However, the patients with moderate to severe depression did not exhibit any meaningful alterations in the olfactory mucosal miR-206 level, regardless of their clinical cognitive impairments. Neuropsychiatric symptoms are common in dementia^46^, and cognitive function can be impaired in severely depressed patients (termed pseudodementia)^47^. Differentiating between these two conditions will be very helpful in clinical practice because it will prevent patients with depression from being prescribed dementia drugs.

Fourth, the olfactory mucosal miR-206 level can be assessed easily without complications and at a low cost. No serious side effects occurred as a result of the 41 OE biopsies performed in our study, and <10% of the participants complained of minor side effects. One commonly used imaging biomarker for AD is the Pittsburgh compound B-PET or FDG-PET, which is quite costly. OE biopsy and real-time PCR can be performed at a much lower cost than PET. Excluding exposure to radioactive isotopes, PET studies require many prior steps before imaging is conducted, which can result in a total study time of several hours^48^. Conversely, olfactory biopsy takes less than 30 minutes and can be performed in an outpatient department by an otolaryngologist alone.

Lastly, measurement of the olfactory mucosal miR-206 level may aid in the selection of proper candidates for precision medicine in AD treatment. Currently, many anticancer treatments are tailored according to the specific mutation carried by the patient^49^,^50^. Precision medicine has recently been attracting increasing attention for its potential to overcome the biological complexity of neurodegenerative diseases, such as AD and Parkinson’s disease^51^,^52^. miRNA-based therapeutics are an excellent example of individualized interventions. If we can identify an overexpressed miRNA that plays a central role in the pathogenesis of a disease, then we can administer an antagomir, which is a complementary sequence to a certain miRNA, to neutralize the effect of the elevated miRNA level^53^. Miravirsen (Santaris Pharma), a drug used in hepatitis C treatment, is the most advanced miRNA-based drug. This agent inhibits miR-122 in the liver, and its evaluation in a phase 2 trial has been successfully completed^54^. We have previously demonstrated the therapeutic effect of antagomir-206 in an AD mouse model^17^. As we can now identify patients with an elevated miR-206 level in the brain by OE biopsy, we can consider antagomir-206 treatment for these patients. The therapeutic potential of antagomir-206 in ‘miR-206-elevated’ dementia patients is very promising, and this tailored approach would represent a breakthrough in AD drug development.

To avoid misinterpretation of the olfactory mucosal miR-206 level, it must be kept in mind that several other conditions are known to be associated with a dysregulated miR-206 level in the brain. Usually, miR-206 appears to be present at an undetectable or very low level in normal brains, except in the cerebellum^55^. However, upregulation of miR-206 has been reported in animal models of cerebral ischemia^56^ and alcohol dependence^34^. Patients with amyotrophic lateral sclerosis have elevated miR-206 expression in the frontal cortex^57^. Therefore, when these conditions are combined with cognitive impairment, it may affect the diagnostic efficacy of olfactory mucosal miR-206, which should be interpreted with caution.

It needs to be investigated in the future whether other miRNAs, which have been reported to be upregulated in the brains of AD patients (i.e. miR-146a, miR-125b, and miR-9)^58^,^59^, are also elevated in the OE of AD patients. However, considering that OE is a known source of BDNF production^60^ and the BDNF expression in OE is dynamically regulated^61^, we assume that miR-206 plays the essential role among the miRNAs and therefore it would be an excellent biomarker of early AD.

Although the current study includes a relatively small number of patients and the classifications of MCI and AD were purely based on the use of clinical scales and not current biomarkers (i.e., PET imaging, atrophy on MRI, or CSF markers), we suggest that the olfactory mucosal miR-206 level is a promising biomarker of early AD. It carries greater sensitivity and specificity than previous AD biomarkers and can detect patients in the MCI stage. Moreover, it enables clinicians to differentiate cognitive decline caused by severe depression from early AD. Large-scale studies are required to validate the efficacy and safety of the procedure. These studies will validate the cutoff values for MCI and early AD. Tailored drug trials targeting ‘miR-206-elevated’ dementia patients are promising and should be conducted in the near future.

## Methods

### Patient recruitment and clinical evaluation

The institutional review board of Seoul National University Hospital approved the study protocol, and informed consent was obtained from all participants. The study was carried out in accordance with the approved guidelines. Subjects over 50 years of age were included in the study. Patients with cognitive impairment were recruited from the neurology outpatient clinic of Seoul National University Hospital, and participants with normal cognitive function were recruited by advertisement. To assess cognitive function, each patient was assessed using the CDR scale, MMSE-K, and ADAS-cog-K. BDI-II was also used to examine each participant to identify the cognitive impairment caused by depression. All clinical assessments were performed by an experienced researcher (J.Y. Shim) on the same day as intranasal biopsy. Patients with moderate to severe depression (BDI-II ≥20) were assigned to the depression group regardless of their cognitive function scores. Patients who met the criteria for probable Alzheimer’s disease of the National Institute of Neurological and Communicative Disorders and Stroke and the Alzheimer’s Disease and Related Disorders Association^4^ were assigned to the dementia group (CDR 1), and those with cognitive impairment without a clinical diagnosis of dementia were assigned to the MCI group (CDR 0.5). People with normal cognitive function (CDR 0) were placed in the control group. The baseline olfactory function was assessed using the KVSS.

### Intranasal biopsy of the olfactory epithelium

Intranasal biopsy was performed at Seoul National University Hospital Otolaryngology clinic as previously described, with some modifications^35^,^39^. A cotton pledget soaked in a mixture of 2% lidocaine and 0.1% epinephrine was inserted into the olfactory cleft for vasoconstriction and local anesthesia. The biopsy procedure was performed by an experienced otolaryngologist (I.G. Kong) under endoscopic guidance. Tissue samples were obtained from the anterior nasal septum, from a location as high as possible, just medial to the insertion of the middle turbinate. Using a small curette or small nasal biting forceps, two or three 2-mm tissue blocks were obtained from one nostril. Nasal packing was performed for 10 minutes after biopsy to control bleeding. The subjects were observed in the clinic for 15 to 30 minutes and then discharged. Tissue donors were informed not to blow their nose and to avoid heavy straining for 24 h. All but 3 patients underwent KVSS before biopsy to evaluate baseline olfactory function.

### Quantitative real-time PCR of miR-206

Total RNA was isolated from the individual nasal mucosal tissue samples with Trizol (Invitrogen, USA), and miRNA was isolated using an Ambion mirVana Isolation Kit. Real-time PCR was performed using a mirVana qRT-PCR miRNA Detection Kit with primer sets for miR-206 and U6 snRNA (Applied Biosystems, USA). All real-time PCR reactions were conducted in triplicate with an ABI PRISM 7000 sequence detection system (Applied Biosystems, USA), and 40 cycles of amplification were performed. Relative expression was calculated using the comparative threshold cycle method, and the miRNA levels were normalized to the U6 levels.

### Statistical analysis

Comparisons between groups were performed using Student’s t-test or the Kruskal-Wallis test. Correlations between variables were calculated using the Spearman rank correlation test, and linear logistic regression analysis was performed. ROC curves were generated to determine the best cutoff value of the relative miR-206 level to detect patients with CDR 0.5 or CDR 1 dementia. The optimal cutoff value was defined as the highest sum of the sensitivity and specificity, and the AUC was analyzed. The 95% CIs for sensitivity and specificity were derived from the Wilson score method^62^. All statistical analyses were performed using SPSS 21.0 for Windows (SPSS, Inc., USA), and the results were considered significant at a P < 0.05.

## Additional Information

**How to cite this article**: Moon, J. *et al*. Early diagnosis of Alzheimer’s disease from elevated olfactory mucosal miR-206 level. *Sci. Rep*. **6**, 20364; doi: 10.1038/srep20364 (2016).

## Supplementary Material

Supplementary Information

## Figures and Tables

**Figure 1:**
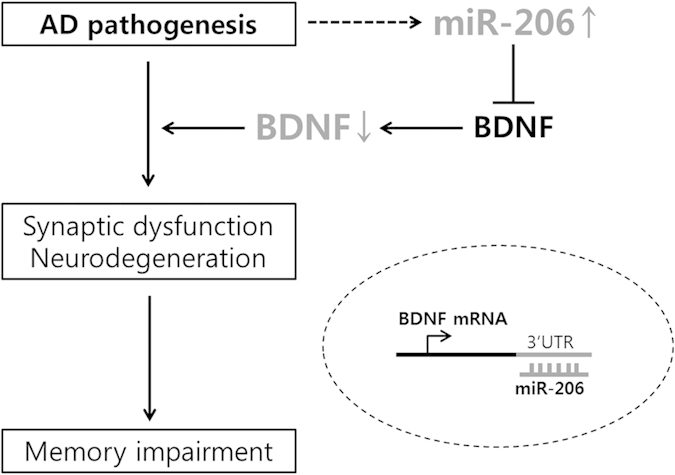
A schematic representation of the role of miRNA-206 in Alzheimer’s disease. Abbreviations: AD: Alzheimer’s disease, miR: microRNA, BDNF: brain-derived neurotrophic factor, mRNA: messenger RNA, 3′ UTR: 3′ untranslated region.

**Table 1:** The basic demographics and clinical assessment scores of the patients (n = 41).

	CDR 0 (n = 9)	CDR 0.5 (n = 13)	CDR 1 (n = 11)	Depression (n = 8)	P-value†
Age, median (range)*	63 (50–74)	68 (56–79)	69 (57–80)	61.5 (56–66)	0.039
Sex (M:F)	4:5	4:9	6:5	2:6	
MMSE-K	28.3 ± 0.7	26.8 ± 0.4	23.8 ± 0.6^a,d^	25.1 ± 1.4	0.001
CDR	0	0.5	1	0.6 ± 0.2	
ADAS-Cog-K	8.4 ± 1.4	11.2 ± 1.1	16.0 ± 2.4^c^	14.6 ± 3.2	0.031
BDI-II	5.2 ± 1.8	11.1 ± 1.4^c^	9.3 ± 2.0	29.4 ± 2.4^a,d,f^	<0.001
KVSS	24.2 ± 1.1	23.6 ± 1.6	22.6 ± 0.9	21.9 ± 2.6	0.750
Relative miR-206 level‡	1	7.8 ± 1.9^b^	41.5 ± 6.4^a,d^	1.2 ± 0.5^e,f^	<0.001

^*^Data presented as the median (range).

^†^The Kruskal-Wallis test was performed.

^‡^The relative miR-206 levels are divided by the mean value for the CDR 0 group.

^a^P < 0.001 vs. control.

^b^P < 0.01 vs. control.

^c^P < 0.05 vs. control.

^d^P < 0.001 vs. CDR 0.5.

^e^P < 0.01 vs. CDR 0.5.

^f^P < 0.001 vs. CDR 1.

Values are presented as the mean ± standard error of the mean (SEM). Abbreviations: CDR: clinical dementia rating, M: male, F: female, MMSE-K: Korean version of the Mini-Mental State Examination, ADAS-Cog-K: Korean version of the Alzheimer’s Disease Assessment Scale-cognitive subscale, BDI-II: Beck Depression Inventory-II.
