# Supplementary material for: Early diagnosis of Alzheimer’s disease from elevated olfactory mucosal miR-206 level
Source: Sci Rep. 2016 Feb 4;6:20364. doi: 10.1038/srep20364 (PMC4740889; doi:10.1038/srep20364)
Supplement: Supplementary Information [file srep20364-s1.pdf]

Supplementary Information

Early diagnosis of Alzheimer's disease from elevated olfactory mucosal miR-206 level

Authors: Jangsup Moon, Soon-Tae Lee, Il Gyu Kong, Jung-Ick Byun, Jun-Sang Sunwoo, Jung-Won Shin, Ji-Young Shim, Ji-Hyun Park, Daejong Jeon, Keun-Hwa Jung, Ki-Young Jung, Dong-Young Kim, Sang Kun Lee, Manho Kim, Kon Chu

Supplementary Table S1. The clinical features and detailed test results for the individual patients

Group	Sex	Age	BDI-2	MMSE	CDR	ADAS-Cog	KVSS	ΔCт Mean	2^-dCT	2^-dCT*10^4	Complication
CDR 0	F	74	13	28	0	9.1	21.5	10.59588	0.00065	6.46132	none
CDR 0	M	68	1	29	0	14.3	18	10.92919	0.00051	5.12844	none
CDR 0	F	57	7	30	0	4.3	26.5	12.75072	0.00015	1.45094	none
CDR 0	M	69	2	23	0	13.6	25.5	12.70721	0.00015	1.49537	none
CDR 0	M	50	12	30	0	5.8	25	11.16770	0.00043	4.34699	mild headache
CDR 0	F	53	2	29	0	5.3	23	10.90108	0.00052	5.22934	none
CDR 0	M	56	0	29	0	11.5	26.5	12.36474	0.00019	1.89602	sustained nasal bleeding
CDR 0	F	63	0	29	0	8.1	29	13.76389	0.00007	0.71888	none
CDR 0	F	68	10	28	0	3.3	23	12.68512	0.00015	1.51845	none
CDR 0.5	M	72	12	27	0.5	17	20	9.64579	0.00125	12.48328	none
CDR 0.5	F	77	15	28	0.5	11.2	20.5	11.13268	0.00045	4.45377	none
CDR 0.5	M	59	12	27	0.5	16.8	31	10.15675	0.00088	8.76019	none
CDR 0.5	F	68	17	26	0.5	8.5	26	11.25944	0.00041	4.07915	none
CDR 0.5	F	79	6	26	0.5	15.3	14	10.42393	0.00073	7.27922	none
CDR 0.5	F	60	17	27	0.5	9.5	27	11.31338	0.00039	3.92946	none
CDR 0.5	M	68	4	27	0.5	10.1	24.5	8.66572	0.00246	24.62390	sustained nasal bleeding
CDR 0.5	F	60	4	26	0.5	15.1	28.5	7.31626	0.00627	62.74625	none
CDR 0.5	F	68	19	25	0.5	13.5	26.5	7.36423	0.00607	60.69403	none
CDR 0.5	F	78	12	29	0.5	8.1	26	8.17483	0.00346	34.60431	none
CDR 0.5	F	63	11	27	0.5	8.8	30.5	7.81144	0.00445	44.51657	none
CDR 0.5	M	56	11	29	0.5	6.6	15.5	7.89069	0.00421	42.13731	none
CDR 0.5	F	66	4	24	0.5	5.5	17	10.21946	0.00084	8.38757	none

CDR 1	M	75	3	26	1	12.5	24.5	6.36321	0.01215	121.47419	none
CDR 1	M	60	19	21	1	18.1	18	5.55832	0.02122	212.21689	none
CDR 1	F	71	19	22	1	18.8	26	5.93141	0.01639	163.85759	none
CDR 1	F	57	10	26	1	9.6	24	6.64777	0.00997	99.72909	none
CDR 1	F	69	18	22	1	12	21	5.33047	0.02485	248.52375	none
CDR 1	M	68	7	26	1	17.8	N/A	5.90438	0.01670	166.95730	none
CDR 1	F	80	3	21	1	36.5	18.5	7.92495	0.00411	41.14842	none
CDR 1	F	80	7	24	1	14	26	6.38595	0.01196	119.57435	none
CDR 1	M	66	6	25	1	6	N/A	6.06499	0.01494	149.36778	none
CDR 1	M	68	9	26	1	16.6	22.5	7.50821	0.00549	54.92910	none
CDR 1	M	74	1	23	1	14.3	N/A	7.47595	0.00562	56.17148	none
Depression	F	64	30	28	0.5	8.3	24.5	14.75719	0.00004	0.36111	none
Depression	M	56	44	22	1	34.3	21.5	13.56191	0.00008	0.82691	none
Depression	F	59	29	17	1	20.3	20	11.05978	0.00047	4.68461	none
Depression	F	64	33	26	1	10.6	6	11.75768	0.00029	2.88792	none
Depression	F	62	28	28	0	8.5	29	10.51300	0.00068	6.84340	none
Depression	F	61	25	24	1	13.8	27.5	12.94812	0.00013	1.26540	watery rhinorrhea
Depression	F	66	24	27	0.5	13.1	20	9.64357	0.00125	12.50250	none
Depression	M	59	22	29	0	8	26.5	13.04149	0.00012	1.18610	none

Supplementary Table S2. The relative miR-206 levels in each age group

**Relative miR-206 level†**	**CDR 0**	**CDR 0.5**	**CDR 1**	**Depression**	**P-value***
< 65 years old (n=19)	1	11.9±4.1[b]	57.1±20.6[a,d]	0.9±0.3[e]	0.011
> 65 years old (n=22)	1	5.4±1.9	34.1±6.1[a,c]	3.4	0.001

Values are presented as the mean±standard error of the mean (SEM).

* The Kruskal-Wallis test was performed.

† The relative miR-206 levels are divided by the mean value for the CDR 0 group.

[a] P < 0.01 vs. control

[b] P < 0.05 vs. control

[c] P < 0.001 vs. CDR 0.5

[d] P < 0.05 vs. CDR 0.5

[e] P < 0.001 vs. CDR 1

Abbreviations: CDR: clinical dementia rating

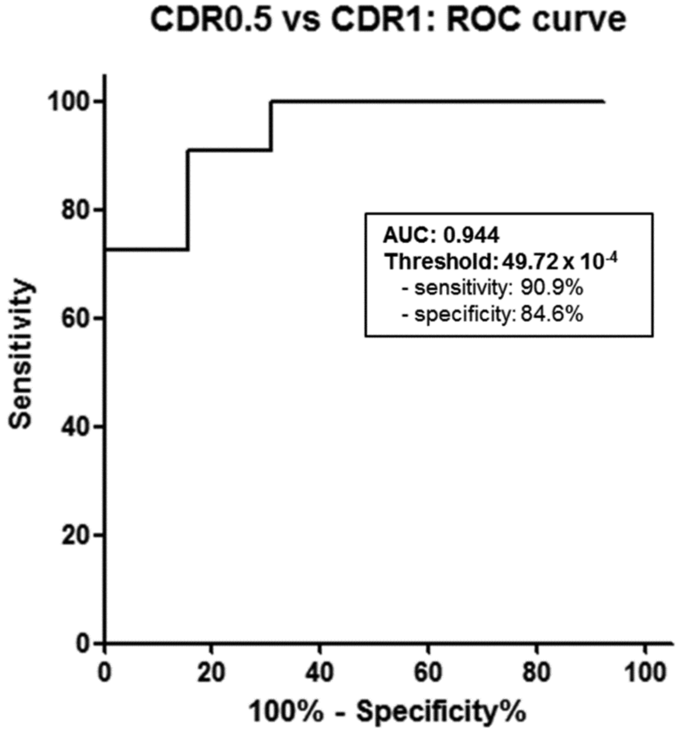

Supplementary Figure S1. Receiver operating curve analysis of non-depressed cognitive impairment patients

The non-depressed cognitive impairment patients were selected out, and receiver operating curve (ROC) analysis was performed. The area under the curve (AUC) is 0.944, and the sensitivity and specificity for diagnosing CDR 1 dementia are 90.9% and 84.6% at the optimal cutoff value.

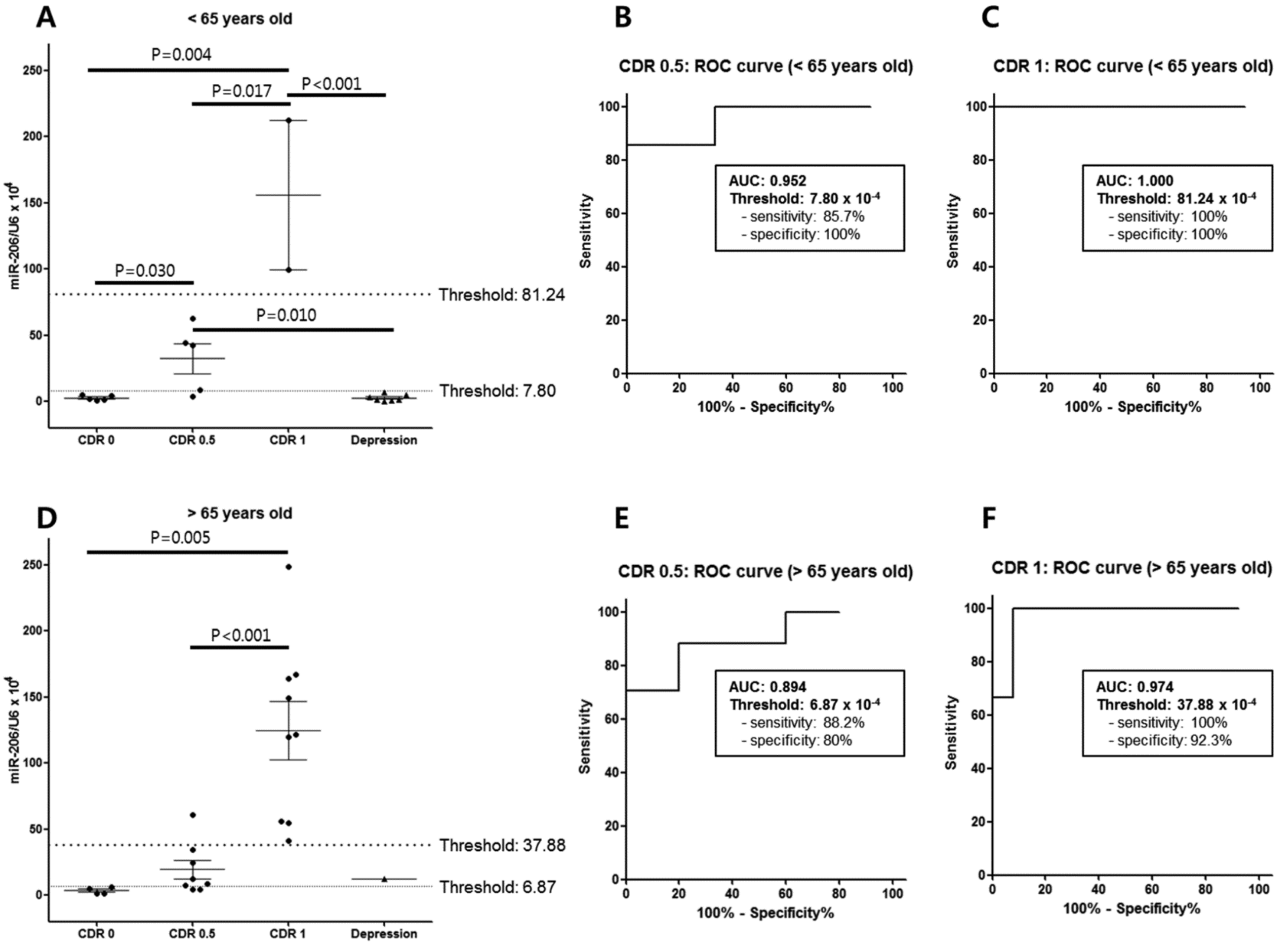

Supplementary Figure S2. miRNA-206 real-time PCR results for olfactory epithelial samples from different age groups (< 65 years old versus > 65 years old)

Panels A-C demonstrate the relative miRNA-206 level (normalized to the U6 level) and receiver operating curve (ROC) analysis results for the patients less than 65 years old. The areas under the curve (AUCs) are 0.952 and 1.000 for CDR 0.5 and CDR 1 dementia, respectively. Panels D-F demonstrate the relative miRNA-206 level and ROC analysis results for the patients older than age 65. The AUCs are 0.894 and 0.941 for CDR 0.5 and CDR 1 dementia, respectively.
